# Effect of Pentoxifylline on Microalbuminuria in Diabetic Patients: A Randomized Controlled Trial

**DOI:** 10.1155/2015/259592

**Published:** 2015-03-22

**Authors:** Shahrzad Shahidi, Marziyeh Hoseinbalam, Bijan Iraj, Mojtaba Akbari

**Affiliations:** ^1^Isfahan Kidney Disease Research Center, Isfahan University of Medical Sciences, Isfahan, Iran; ^2^Isfahan Endocrine and Metabolism Research Center, Isfahan University of Medical Sciences, Isfahan, Iran; ^3^Department of Epidemiology, School of Health and Nutrition, Shiraz University of Medical Sciences, Shiraz, Iran

## Abstract

*Background*. Pentoxifylline is a nonspecific phosphodiesterase inhibitor with anti-inflammatory properties. Human studies have proved its antiproteinuric effect in patients with glomerular diseases, but this study was designed to assess the effects of add-on pentoxifylline to available treatment on reduction of microalbuminuria in diabetic patients without glomerular diseases. *Methods*. In a double-blind placebo-controlled, randomized study we evaluated the influence of pentoxifylline on microalbuminuria in type 2 diabetic patients. 40 diabetic patients with estimated glomerular filtration rate (eGFR) of more than 60 mL/min/1.73 m^2^ in eight weeks and microalbuminuria were randomized to two groups which will receive pentoxifylline 1200 mg/day or placebo added to regular medications for 6 months. albuminuria; eGFR was evaluated at three- and six-month follow-up period. *Results*. Baseline characteristics were similar between the two groups. At six months, the mean estimated GFR and albuminuria were not different between two groups at 3- and 6-month follow-up. Trend of albumin to creatinine ratio, systolic and diastolic blood pressure, and eGFR in both groups were decreased, but no significant differences were noted between two groups (*P* value > 0.05). *Conclusion*. Pentoxifylline has not a significant additive antimicroalbuminuric effect compared with placebo in patients with type 2 diabetes with early stage of kidney disease; however, further clinical investigations are necessary to be done.

## 1. Introduction

Diabetes is among the most common and major diseases in the world and recently in most countries the number of patients with diabetes has strikingly increased. Diabetic nephropathy is enlisted as one of the chronic microvascular complications of diabetes which is associated with considerable morbidity and mortality [1, 2] and is a main cause for approximately 50% of all end stage renal disease, and this results in increasing renal replacement therapy and healthcare costs [3, 4]. Though many pathophysiologic processes are involved in the pathogenesis of diabetic nephropathy, the fundamental mechanisms of it are not fully established [5]. Diabetic nephropathy is characterized by proteinuria, hypertension, and advanced renal insufficiency. More than 350 million people will be afflicted by diabetes by 2030 [6]; and about 20 to 30 percent of these diabetic patients, either type 1 or type 2, will be suffering from diabetic nephropathy, which has a greater incidence as the disease becomes more chronic [7].

Recently, the focus has moved to much earlier stages in renal disease as established by the presence of microalbuminuria [8, 9] and this is an early sign of diabetic nephropathy and premature cardiovascular disease [9, 10]. Biannual control of microalbuminuria in patients with diabetes is recommended in American and European guidelines [11, 12]. Microalbuminuria indicates a possibility of ongoing renal involvement due to diabetic nephropathy which ultimately results in end stage renal disease [13]. In type 2 diabetes, microalbuminuria or overt proteinuria may be present by the time of diagnosis and the latter is often accompanied by hypertension in these patients; however conditions such as congestive heart failure, hypertension, and infections can also lead to microalbuminuria in diabetic patients [2, 14]. It is reported that approximately a total of 3.7 percent of type 2 diabetic patients go toward advanced renal complications, and the risk for major renal involvements in patients with microalbuminuria is two times greater than normoalbuminuric patients [15]. This reinforces the necessity of early detection and treatment of microalbuminuria.

One effective treatment is the use of angiotensin converting enzyme inhibitor (ACEI) which delivers its effect in delaying the development of diabetic nephropathy through inhibition of renin-angiotensin system; however these treatments are being shown to be not only time consuming, but also not preventive enough. Studies have shown that beside metabolic and hemodynamic changes, inflammatory phenomena are also involved in progression of diabetic nephropathy, implying that the anti-inflammatory drugs could be beneficial [16–18].

Pentoxifylline is a nonselective phosphodiesterase inhibitor that is used in peripheral vascular diseases. There have been several theories for its mechanism of action, including anti-cell proliferation, and being anti-inflammatory and anti-fibrotic [19–22]. Clinically, pentoxifylline has been shown to be beneficial in nephropathies by reducing proteinuria and TNF-*α* level; however its overall advantage for nephropathies is still a matter of debate [23–25]. Few studies have shown that coapplication of pentoxifylline with ACEI drugs in diabetic patients or its use together with immunosuppressant drugs in nephrotic syndrome of lupus patients resulted in a reduction of proteinuria [26, 27]. It is yet not clear whether or not pentoxifylline is effective on reduction of proteinuria. So, the present study was designed to assess the effects of pentoxifylline on microalbuminuria in diabetic patients.

## 2. Materials and Methods

The present study was a randomized, parallel-group, double blind study which was conducted between Sep. 2012 and Sep. 2013, on 50 adult patients with diabetic nephropathy in our city endocrine and metabolism research center outpatient clinic. The ethics committee of our University of Medical Sciences approved the study. Eligibility was defining as age older than 18 years in both genders, with an estimated glomerular filtration rate (GFR) higher than 60 mL/min/1.73 m^2^ in the last six months. GFR was estimated using the 4-variable Modification of Diet in Renal Disease (MDRD). Also, patients were screened only if blood pressure (BP) was less than 140/90 mm Hg by using beta blockers, ACEI or ARB, without pressure alterations, under interventions, on a diet with protein intake levels less than 0.8 gr/kg/d, and glycosylated hemoglobin (HbA1c) less than 8%. Patients were eligible for enrollment if spot urine albumin-creatinine ratio was less than 300 mg/g in 3 consecutive measurements during a 3-month screening period.

Patients were excluded if they had myocardial infarction, had undergone coronary artery bypass grafting or percutaneous transluminal coronary angioplasty, or had a stroke or a retinal hemorrhage within the prior 6 months. Additional exclusion criteria included abnormal liver function test results, congestive heart failure (New York Heart Association class III or IV), obstructive uropathy, active malignancy, and being not able to discontinue immunosuppressive or nonsteroidal anti-inflammatory drugs, as well as pregnant women or those who do breast feeding, or any changes in patient's medications during the examination on any basis including high blood pressure and patient's disinclination for enrollment or intolerance to pentoxifylline.

After obtaining written informed consent from studied patients, all participating patients underwent primary examinations by a physician. Age, sex, weight, height, systolic and diastolic blood pressure, GFR (based on the MDRD formula), and urinary albumin/creatinine ratio in a morning spot urine sample were measured and calculated. Then, using random-maker software “Random Allocation” eligible patients were randomly divided into two 20-member groups. Case group includes patients who received 400 mg pentoxifylline pills (manufactured by Amin Pharmaceutical, three times a day) besides the patients' regular medications, and control group includes patients who received placebo similar to pentoxifylline pills besides the patients' regular medications. During the study patients' blood pressure was monitored and controlled via their routine monthly examinations by their physicians; no changes were made in neither the type nor the dosage of their hypertensive drugs. Also, three and six months later, the participants' albumin/creatinine ratio in spot urine sample, systolic and diastolic blood pressure, and GFR were measured.

All statistical analyses were done using SPSS software for Windows, version 20. Descriptive data are reported as mean ± SD, or number (percent) as appropriate. Independent Samples Test and Chi-square test and ANOVA were used for comparing all studied variables between groups as appropriate. The level of significance is considered to be less than 0.05.

## 3. Results

**Figure 1:**
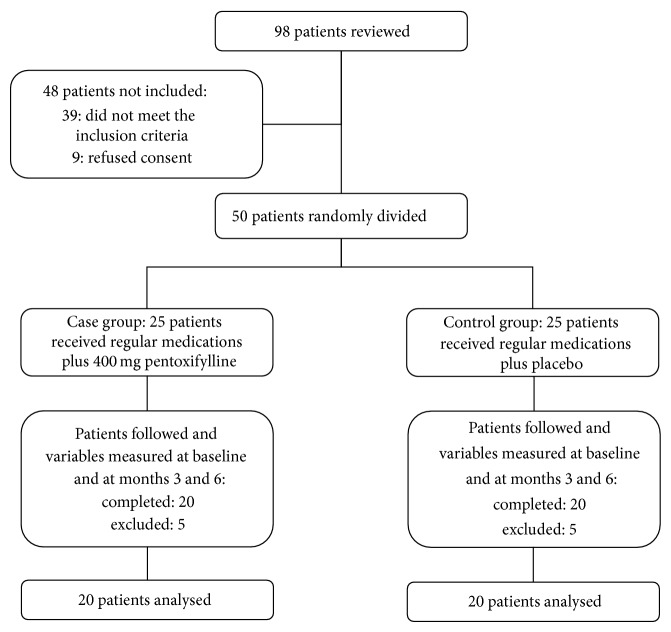
Patients who entered to the study, divided into the study groups and analyzed.

Of 98 reviewed patients, 48 patients did not enter to the study (39 patients were not eligible and nine patients refused informed consent). Fifty patients were eligible and randomly assigned into two treatment groups. Patients were followed up for 6 months. During the follow-up, 10 patients (5 patients in control and 5 patients in case groups) in both groups were excluded due to gastrointestinal problems and, finally, 40 patients in both groups completed the study and were analyzed (Figure 1).

The mean age of the studied patients was 53.2 ± 10.2 years; 25 patients (62.5%) were female and 15 patients (37.5%) were male.  Table 1 shows baseline characteristics of studied patients. No significant differences were noted between case and control groups for mean of age and sex combination, weight, GFR, duration of being diabetic, BMI, and HbA1C (*P* value > 0.05).

**Figure 2:**
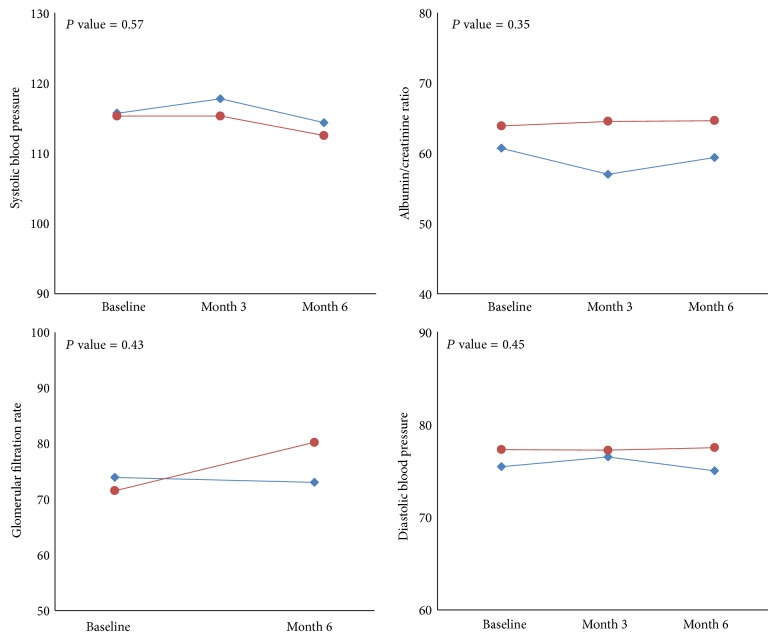
Comparison of trend of studied variables in time points between case and control groups. Case group included 20 patients who received regular medications plus 400 mg pentoxifylline. Control group included 20 patients who received regular medications plus placebo. Statistical analyses were done using ANOVA and no significant differences were noted between groups for trend of albumin/creatinine ratio, systolic and diastolic blood pressure, and glomerular filtration rate at time points (*P* values > 0.05).

Table 2 shows the comparison of patients' ratio of albumin to creatinine between study groups, systolic and diastolic blood pressure, and GFR at studied time points. As shown the ratio of albumin to creatinine was similar between two groups and no significant difference was noted between cases and controls (*P* value > 0.05). Patients' systolic and diastolic blood pressure and GFR at studied time points, in both case and control groups, were not statistically significantly different (*P* value > 0.05). Also trend of ratio of albumin to creatinine, systolic and diastolic blood pressure, and GFR between groups during six-month follow-up were analyzed by ANOVA and results are reported in Figure 2. As shown trend of ratio of albumin to creatinine, systolic and diastolic blood pressure, and GFR in both groups were similar and no significant differences were noted between groups (*P* values > 0.05).

Drugs used by patients before and during study period were reported in all patients (Table 3) including eight patients in pentoxifylline group and 11 patients in control group for ARBs (*P* value = 0.12), three of patients in pentoxifylline group and two patients in control group for ACEI (*P* value = 0.81), two patients in pentoxifylline groups and one in control group for *β*-blocker (*P* value = 0.67), and two patients in both pentoxifylline and control groups for Ca-blocker (*P* value = 0.83). Also, four patients in both pentoxifylline and control groups reported use of diuretic (*P* value = 0.68), and 17 in pentoxifylline group and 14 patients in control group reported use of statin (*P* value = 0.97).

## 4. Discussion

Microalbuminuria is one of the first clinical symptoms of diabetic nephropathy that may progress to macroalbuminuria and the progressive loss of glomerular filtration rate and finally the end stage renal disease [2]. Early recognition and treatment of microalbuminuria can prevent irreversible complications such as kidney problems [10]. Antihypertensive treatments, renoprotective treatments such as angiotensin converting enzyme inhibitors or angiotensin II receptor blockers, antihyperlipidemia medications, and protein intake restriction are treatments that have been used to control diabetic nephropathy [11, 16, 17, 28, 29]. Due to report of some side effects, in all of the diabetic patients these treatments cannot always be used with the proper dosage of the medications and different medications have been assessed in these patients. The present study was undertaken to answer the question whether add-on pentoxifylline to available treatment in diabetic patients would improve microalbuminuria, and we found that the add-on pentoxifylline to available treatment does not decrease microalbuminuria during six-month treatment in these patients compared with placebo. Also differences in the mean of albumin/creatinine ratio of random urine sample and urine creatinine level before and after treatment between groups were not statistically significant. Totally our results showed that add-on pentoxifylline to available treatment does not provide additive antimicroalbuminuric effects in patients with early stage of diabetic nephropathy (stages 1 to 3).

Pentoxifylline is one of a number of anti-inflammatory drugs that have been used for clinical trials in diabetic patients with nephropathy. Effect of pentoxifylline on albuminuria among these has been evaluated in several studies with different findings. Some studies have clearly shown significant decrease in albuminuria in the pentoxifylline group compared with placebo or routine treatment. In a prospective and randomized study, Navarro et al. showed an additional effect on the reduction of urinary albumin excretion of treatment with pentoxifylline in a group of patients with type 2 diabetes and diabetic nephropathy and residual albuminuria despite long-term therapy with angiotensin II receptor blockers at the recommended dosage [27]. Rodríguez-Morán et al. [30] have also compared the efficacy of pentoxifylline and captopril on the reduction of albuminuria in 130 normotensive diabetic patients with normal renal function and concluded that pentoxifylline is an effective alternative agent to ACE inhibitors in reducing albuminuria. In the study of Solerte et al., 21 diabetic patients received pentoxifylline and results showed a significant reduction of albuminuria and arterial blood pressure in these patients compared to other groups with treatment with antihypertensive drugs [31]. Another study by Harmankaya et al. was done on 25 hypertensive type 2 diabetic patients with persistent microalbuminuria and normal renal function under treatment with combining pentoxifylline with an angiotensin converting enzyme inhibitor, lisinopril, on urinary albumin excretion compared with those obtained in a control group of 25 type 2 diabetic patients treated with lisinopril only and reporting a significant reduction in urinary albumin excretion in pentoxifylline group [32]. All the results in these studies concluded that pentoxifylline is an effective alternative agent for reducing albuminuria. Results of the present study were different from these findings and reported no significant reduction in albuminuria in studied patients after add-on pentoxifylline to available treatment compared to placebo. The reason for differences between these studies could be justified by the different medications; whereas in some studies patients were treated only with pentoxifylline, in some other studies some other different medications were included. Other possible reasons of differences between findings are differences in doses of pentoxifylline, time of patients' follow-up, or lack of placebo group.

Currently, to treat peripheral vascular and bronchoconstrictive diseases pentoxifylline is used. Various conditions such as antiphospholipid syndrome, alcoholic hepatitis, and wound healing have a role in the determination of pentoxifylline effects [33, 34]. But in patients with diabetic nephropathy the effects of pentoxifylline are unclear and this might be because of the heterogeneous clinical nature of diabetic nephropathy [35, 36]. It is reported that urinary albumin excretion is the most powerful marker for subsequent renal events in patient with type 2 diabetes and nephropathy and that the degree of albuminuria reduction is linearly related to the subsequent renal protection [37]; thus any further reduction of albuminuria in patients with diabetes is of great importance. Unfortunately, data on this aspect are scarce and additive antimicroalbuminuric effect of pentoxifylline is an interesting question which is sustained over time. Therefore, the reduction of the residual albuminuria by additional approaches needs to be considered.

After excluding 10 patients during follow-up period small sample size is the possible main limitation of the present study. So, future studies with large sample size are suggested to evaluate the effects of body pentoxifylline in patients with diabetic nephropathy.

In conclusion, the results of our study show that, in patients who have type 2 diabetes and are under long-term treatment, pentoxifylline does not have a significant additive antimicroalbuminuric effect compared with placebo; however, further clinical investigation is necessary to determine this effect.

## Figures and Tables

**Table 1:** Baseline characteristics in 40 studies patients by groups.

	Case group (*n* = 20)	Control group (*n* = 20)	*P* value
Age (year)	50.3 ± 9	55.6 ± 10.7	0.1^*^
Sex			
Male	9 (40.9)	6 (33.3)	0.8^†^
Female	13 (59.1)	12 (66.7)	
Weight (kg)	79.60 ± 2.40	78.45 ± 1.68	0.86^*^
Body mass index	30.51 ± 7.13	30.75 ± 4.22	0.90^*^
GFR (mL/min/1.73 m^2^)	73.93 ± 1.05	71.54 ± 9.29	0.45^*^
HbA1C (%)	7.76 ± 1.62	7.4 ± 1.6	0.55^*^
Duration of being diabetic	5.95 ± 5.58	5.54 ± 5.87	0.82^*^

Data expressed as mean ± SD or number (percent). GFR: glomerular filtration rate; HbA1C: glycosylated hemoglobin.

Case group included 20 patients who received regular medications plus 400 mg pentoxifylline. Control group included 20 patients who received regular medications plus placebo.

*P* values calculated by ^*^Independent Samples Test, ^†^Chi-square test.

**Table 2:** Parameters of patients during the study.

	Time points		
	Baseline	Month 3	Month 6
Albumin/creatinine ratio			
Case	60.69 ± 22.04	56.98 ± 25.28	59.37 ± 28.24
Control	63.87 ± 23.26	64.51 ± 31.27	64.63 ± 27.86
*P* value	0.77	0.41	0.58
Systolic blood pressure			
Case	115.68 ± 11.16	117.73 ± 9.72	114.32 ± 11.37
Control	115.28 ± 9.15	115.28 ± 11.17	112.50 ± 11.15
*P* value	0.90	0.46	0.61
Diastolic blood pressure			
Case	75.45 ± 8.15	76.50 ± 5.72	75 ± 5.34
Control	77.29 ± 8.26	77.22 ± 6.69	77.50 ± 8.44
*P* value	0.50	0.88	0.26
Glomerular filtration rate			
Case	73.93 ± 1.05	—	73.05 ± 11.80
Control	71.54 ± 9.29	—	80.22 ± 11.97
*P* value	0.45	—	0.06

Data expressed as mean ± SD.

Case group included patients who received regular medications plus 400 mg pentoxifylline. Control group included patients who received regular medications plus placebo.

*P* values calculated by Independent Samples Test.

**Table 3:** Baseline characteristics in 40 studies patients by groups.

	Case group (*n* = 20)	Control group (*n* = 20)	*P* value
ARB	8 (40)	11 (55)	0.12
ACEI	3 (15)	2 (10)	0.81
Beta-blocker	2 (10)	1 (5)	0.67
Ca blocker	2 (10)	2 (10)	0.83
Diuretics	4 (20)	4 (20)	0.99
Statins	17 (85)	14 (70)	0.97

Data expressed as number (percent). ARB: angiotensin receptor blocker; ACEI: angiotensin converting enzyme inhibitor.

Case group included 20 patients who received regular medications plus 400 mg pentoxifylline. Control group included 20 patients who received regular medications plus placebo.

*P* values calculated by Chi-square test.
